# A generic scope actuation system for flexible endoscopes

**DOI:** 10.1007/s00464-023-10616-7

**Published:** 2023-12-08

**Authors:** Sofia Basha, Mohammad Khorasani, Nihal Abdurahiman, Jhasketan Padhan, Victor Baez, Abdulla Al-Ansari, Panagiotis Tsiamyrtzis, Aaron T. Becker, Nikhil V. Navkar

**Affiliations:** 1 Department of Surgery, Hamad Medical Corporation, Doha, Qatar; 2 Department of Electrical Engineering, University of Houston, Houston, TX USA; 3 Department of Mechanical Engineering, Politecnico Di Milano, Milan, Italy; 4 Department of Statistics, Athens University of Economics and Business, Athens, Greece

**Keywords:** Surgical endoscope, Endoscopy, Actuation, Robotic control, Visualization

## Abstract

**Background:**

A scope actuation system assists a surgeon in steering a scope for navigating an operative field during an interventional or diagnostic procedure. Each system is tailored for a specific surgical procedure. The development of a generic scope actuation system could assist various laparoscopic and endoscopic procedures. This has the potential to reduce the deployment and maintenance costs for a hospital, making it more accessible for clinical usage.

**Methods:**

A modular actuation system (for maneuvering rigid laparoscopes) was adapted to enable incorporation of flexible endoscopes. The design simplifies the installation and disassembly processes. User studies were conducted to assess the ability of the system to focus onto a diagnostic area, and to navigate during a simulated esophagogastroduodenoscopy procedure. During the studies, the endoscope was maneuvered with (*robotic mode*) and without (*manual mode*) the actuation system to navigate the endoscope’s focus on a predefined track.

**Results:**

Results show that the robotic mode performed better than the manual mode on all the measured performance parameters including (a) the total duration to traverse a track, (b) the percentage of time spent outside a track while traversing, and (c) the number of times the scope focus shifts outside the track. Additionally, robotic mode also reduced the perceived workload based on the NASA-TLX scale.

**Conclusions:**

The proposed scope actuation system enhances the maneuverability of flexible endoscopes. It also lays the groundwork for future development of modular and generic scope assistant systems that can be used in both laparoscopic and endoscopic procedures.

Flexible endoscopes are primarily used for diagnostic purposes and are inserted through natural cavities of the human body without the need for any external incisions. An operator maneuvers an endoscope manually using both hands. The left hand is primarily used to control knobs to enable up–down and left–right movement of endoscope’s distal end, whereas the right hand is used to insert-retract and rotate the endoscope along its axis. Proficiency in maneuvering is essential to navigate the endoscope through narrow tubular anatomical structures.

To improve ergonomics and maneuverability, robotic systems have been developed. Initial systems provided shared control where endoscope’s distal end was actuated, and the rest of the endoscope’s movements (i.e., insertion-retraction and rotation) were performed manually by the operator [1–4]. Although these systems assisted in steering the distal end, they required precise coordination between the operator’s hands. This was challenging as it required one hand to provide actuation commands (through an input device), while the other hand was used to manually insert-retract and rotate the endoscope. To address coordination challenges, dedicated robotic systems, each with a unique design, were developed to actuate insertion-retraction and rotation of the endoscopes [5–8]. In the system presented in [5], the distal end of the endoscope shaft is secured between two pre-tensioned wheels. The rotation of the wheels facilitates the process of endoscope insertion/retraction. However, during endoscopy, lubricants are applied, which may potentially decrease the friction and introduce a risk of slippage. Similar issues may occur in the system presented in [6], which requires grasping the endoscope shaft and inserting/retracing it using a mechanized arm. The system presented in [7] performs insertion/retraction and rotation of the endoscope by actuating (translating and rotating) the rear side of the endoscope and guiding it along a rail. The system was primarily developed to show the benefits of eye-gaze control, with less emphasis on ease setup in the Operating Room (OR). As mechanical parts were affixed to the endoscope, it was difficult to switch from robotic to manual mode during the procedure. These aforementioned factors underscore the need to design an operating room-friendly endoscope actuation system.

In our prior work, we developed a generic surgical scope adapter [9–11], capable of hosting different types of rigid laparoscopes, including zero-degree, angulated, and articulated scopes, as well as camera heads. Moreover, it can be connected to any six degree-of-freedom robotic manipulator as an actuation system to maneuver rigid scopes during laparoscopic procedures (Fig. 1a). Building on this foundation, this work extends the aforementioned system to facilitate flexible endoscopic procedures, introducing an endoscopic actuation system (Fig. 1b). The motivation behind developing a modular and versatile system was to enhance its applicability in a wide range of laparoscopic and endoscopic procedures, potentially reducing the cost associated with deploying and maintaining specialized robotic scope actuation systems in hospitals.

**Fig. 1:**
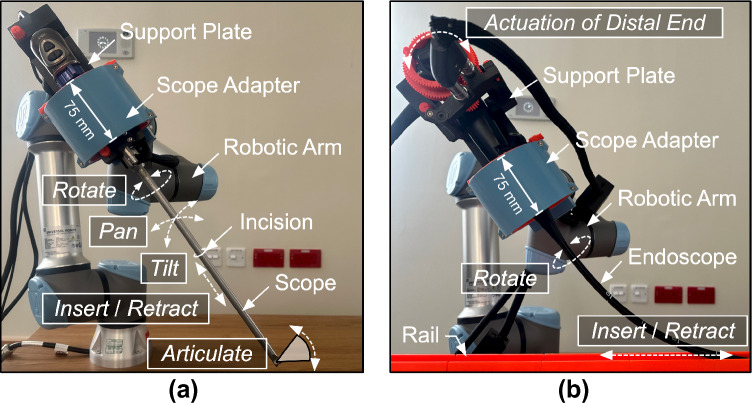
Proposed actuation system designed to support both laparoscopic and endoscopic procedures. A customized support plate (either to host laparoscope or endoscope) is inserted into a scope adapter connected to a robotic arm (UR5e—Universal Robots). **a** Configuration for articulated rigid laparoscope (EndoCAMeleon—Karl Storz). The system facilitates rotation, panning, tilting, insertion/retraction, and articulation of the laparoscope. **b** Configuration for flexible endoscope (EG-590WR—Fujinon). The system allows for rotation and insertion/retraction of the endoscope shaft, as well as two-degree of freedom deflection of the endoscope’s distal end

## Materials and methods

### System design

**Fig. 2:**
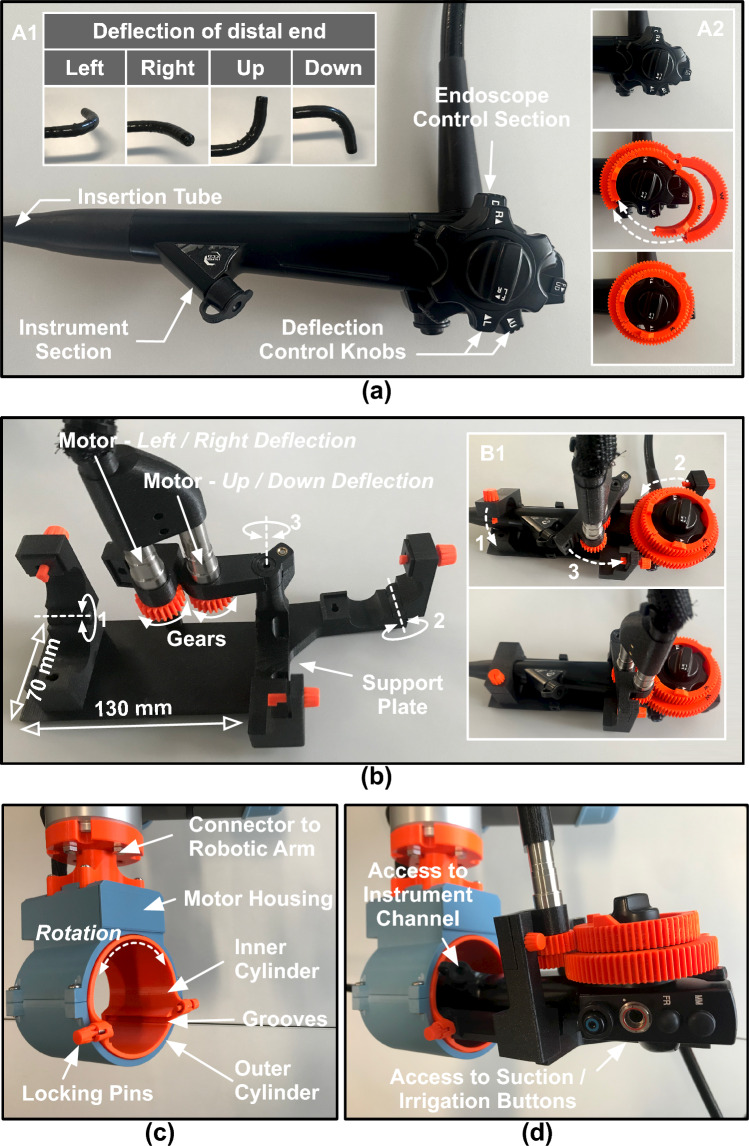
**a** Rear part of an endoscope consisting of control and instrument sections. The control section consists of knobs to deflect the distal end (shown in panel A1). Gears are latched onto these knobs (shown in panel A2). **b** Support plate custom-designed to host an endoscope. An endoscope is affixed onto the support plate using hinges (shown in Panel B1). **c** Design of the scope adapter. **d** Scope adapter hosting a support plate with an endoscope

The design and assembling mechanism of our proposed endoscope actuation system is presented in Fig. 2. An endoscope (for example sigmoidoscope, duodenoscope, gastroscope, and colonoscope) primarily consists of three sections: (a) control section consisting of knobs along with buttons that account for magnification, suction, air, and water supply, (b) instrument section with a channel for passage of diagnostic instruments, and (c) insertion tube with a camera located at the distal end (Fig. 2a). While the former two sections are similar in design, the latter varies with the intervention site. This allowed us to design a generic support plate that could host the rear end of the endoscope (comprising of control and instrument section) and is also compatible with different endoscope types. The left–right and up–down knobs in the control section are used to deflect the distal tip (shown in Panel A1 of Fig. 2a); this changes the viewing direction of the endoscope. Two circular gears are latched onto these knobs (Panel A2 of Fig. 2a). The support plate hosts two motors (Maxon ECX SP13) to enable deflection of the scope distal end. It also consists of three hinges (shown as hinge 1, 2, and 3 in Fig. 2b). Hinges 1 and 2 are used to secure the position of the endoscope onto the support plate, and hinge 3 engages the gears of the motors with the gears latched onto the control knobs (Panel B1 of Fig. 2b).

A scope adapter, developed previously for rigid scopes [9], was then used to host the support plate. The scope adapter consists of two concentric cylinders, where the inner cylinder rotates inside the outer cylinder (Fig. 2c). The support plate is inserted into the inner cylinder along the grooves and secured in position using locking pins. Rotation of the inner cylinder rotates the endoscope shaft. The support plate was designed such that it provided access to the instrument channel and irrigation/suction buttons (Fig. 2d). A connector (shown in Fig. 2d) is used to attach the scope adapter to a robotic arm. The robotic arm places the scope adapter ergonomically and controls the insertion-retraction of the endoscope along a rail (Fig. 1b).

**Fig. 3:**
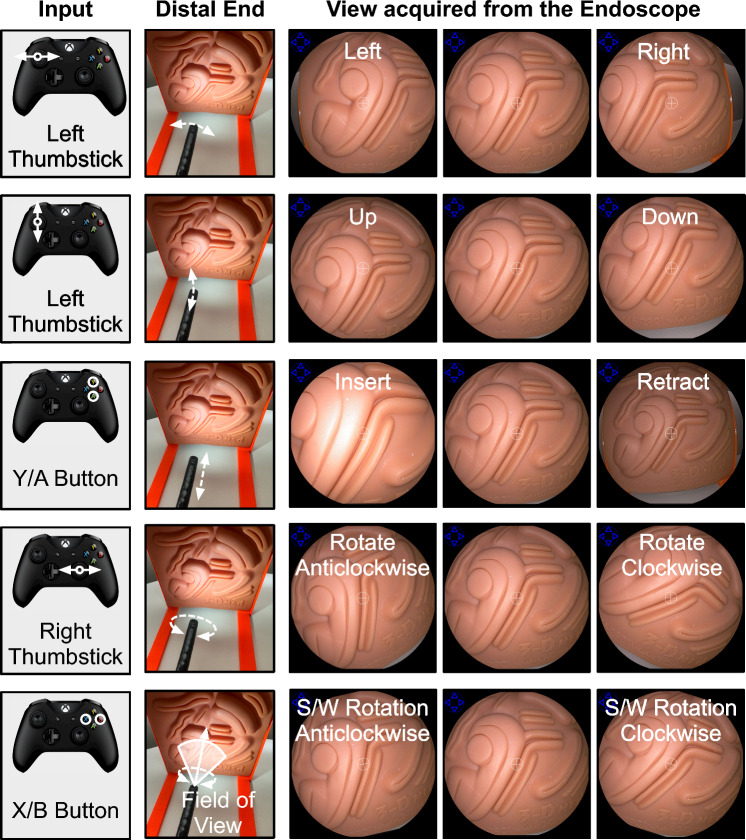
A game controller is used by the operator to provide input commands to the endoscope actuation system. Each command is mapped to a unique movement of the distal end that changes the view acquired from the endoscope. This includes physical movements of the distal end (such as left–right, up–down, insert/retract, and rotation) as well as rotation of the view rendered on the screen

A video-game controller (Xbox controller—Microsoft) is used as the input device to manipulate the five degrees of freedom available to the operator (shown in Fig. 3). The system was actuated only if the thumbsticks were moved out of their neutral position, or the buttons allocated to specific functionality were pressed. This allowed multidirectional motions. The left thumbstick value range was divided into five levels on both sides to allow motor rotating at five different speeds with gradual increment [2]. This implementation enabled faster deflections of the distal tip along straight paths and slower, more controlled deflections along the curved paths. The right thumbstick controls the rotation of the inner cylinder that actuates the rotation of the endoscope along its shaft. The buttons on the controller are used to control (a) the software rotation of the endoscope view rendered on the screen, and (b) translation of the scope adapter along a predefined direction set by the operator for the insertion/retraction of the endoscope along the rail (Fig. 1b).

### System evaluation

#### User study

A user-study was conducted with 10 subjects (aged between 23 and 40 years; 4 females and 6 males) from Department of Surgery at Hamad General Hospital. The subjects engaged for this study were researchers working in the field of medical robotics with in-depth knowledge of surgical scope handling for laparoscopic and endoscopic procedures. The study was approved by institutional review board ethical committee (Medical Research Center, Doha, Qatar, approval number MRC-01-20-087). To ensure subject’s familiarity with the system, a preparatory session lasting 20–30 min was conducted before the commencement of the study. The session aimed at training the basic skills of flexible endoscopy, namely, maneuvering the endoscope tip in both horizontal and vertical directions, employing the push-and-pull technique, and understanding torque application on the endoscope shaft [12]. The session concluded when subjects demonstrated (a) proficiency in mapping the scope movements (tip deflection, insertion-retraction) with manual maneuvers as well as joystick control and (b) visual comprehension of view rendered on the screen. This ensured that the subjects were equally experienced in both modes of actuation. Two scenarios (Scenario-A and Scenario-B) were used to assess the efficacy of the proposed endoscope assistant system. In each scenario, the subject performed the user study under two modes of actuation: (a) ‘*Manual*’ where the subject manually controlled the endoscope (without the proposed system), and (b) ‘*Robotic*’ where the subject used the proposed system to actuate the endoscope.

**Fig. 4:**
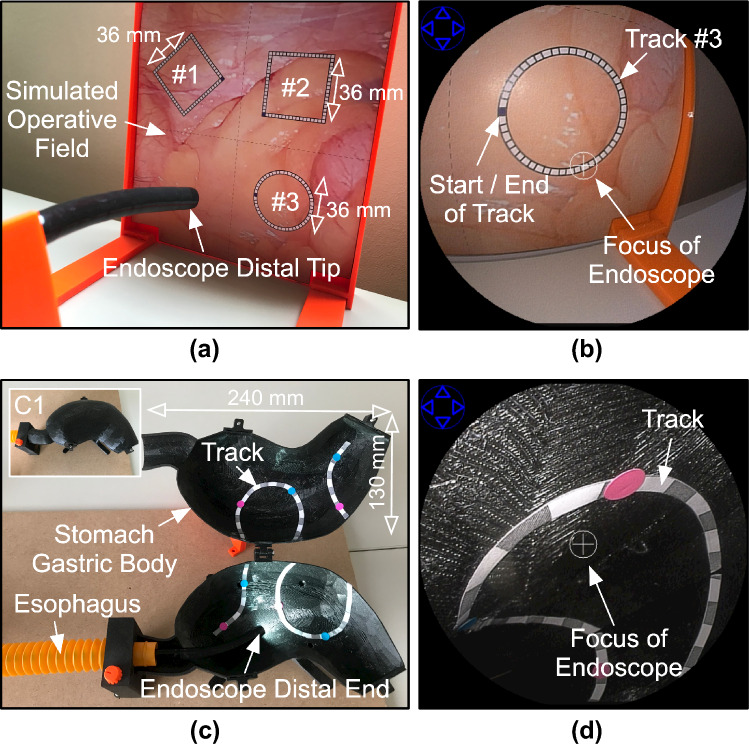
**a** Experimental setup for user study conducted in Scenario-A. **b** View from the endoscope while traversing the track #3 in Scenario-A. **c** Experimental setup employed for the user study in Scenario-B. During the user study, the endoscope is navigated inside a closed stomach phantom (as shown in panel C1). **d** View from the endoscope while traversing the track inside stomach gastric body in Scenario-B

Scenario-A assessed the system’s ability to precisely focus onto an operative field by deflecting the distal end. Only the deflection of the scope distal end was enabled. The software rotation of the view was kept active, if required. The subjects were asked to navigate three tracks (named #1, #2, and #3) drawn on a simulated operative field (shown in Fig. 4a). The tracks were divided into square segments (2 × 2 mm). The subjects were asked to traverse the track from start to end while keeping the focus of the endoscope on the track (shown in Fig. 4b). The focus is shown as a “ + ” sign rendered in the center of the view acquired from the endoscope.

Scenario-B assessed the system’s ability to follow a path using all five endoscope movements inside an organ. A part of an esophagogastroduodenoscopy procedure was simulated [7]. The setup consisted of an esophagus tube (30 cm in length and 3 cm in diameter) and a 3D printed stomach phantom (shown in Fig. 4c). The subjects were asked to navigate the track placed along the lining of the stomach from gastric cardia to pyloric antrum, while keeping the focus of the endoscope on the track (Fig. 4d). The track assisted the subject to traverse circular markers (8 mm in diameter) simulating ulcers.

#### Data analysis

To eliminate the bias produced due to systematic collection of experimental data, a simple randomization approach was adopted [13]. For both scenarios, the video stream of the operative field was recorded, and following parameters were measured: (a) duration to traverse the track by maneuvering the endoscope, (b) percentage of time for which the scope’s focus shifts outside the track, and (c) number of missed segments while traversing the track. At the completion of scenario-B, the subjects also completed a NASA-TLX questionnaire on a scale of 1–10 to assess the mental, physical, temporal, performance, efforts, and frustration demands of the robotic mode as compared to manual mode. These constituted the response variables.

The study resulted in a repeated measure design, where subjects provided multiple measurements. In Scenario A, paired *t*-tests were used to compare the response variables between two modes for the three individual tracks. In Scenario B, mixed effects modeling was used for analyzing the response variables. During analysis, mode of actuation was considered fixed effects, whereas subjects were random effects. Paired *t*-tests were also used to compare the scores on NASA-TLX scale for the two modes. After running the statistical models, we performed a detailed model adequacy check to examine whether any of the assumptions were violated.

## Results

**Fig. 5:**
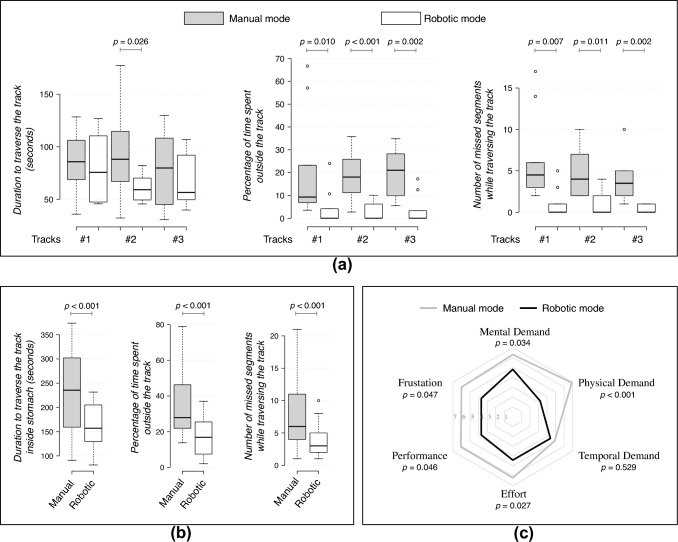
Boxplots presenting the measured parameters to assess the 10-subject user study performed in **a** Scenario-A and **b** Scenario-B. **c** The average scores using the NASA-TXL workload assessment scale (from 1 to 10) for manual and robotic modes in Scenario-B

In Scenario-A, the subjects were able to successfully traverse the tracks under both modes of actuation. The results of the user study are presented in Fig. 5a and Table 1. The duration to traverse track #2 was significantly lower under robotic mode (60.3 ± 11.8 s) compared to manual mode (93.1 ± 38.3 s, *p* = 0.026). There were no significant differences in traversal time observed for track #1 and track #3. However, when compared to the manual mode, robotic mode led to a significant decrease in the percentage of time for which the scope’s focus shifted outside the track. Under manual mode, the percentage of time for which the scope’s focus shifted outside tracks #1, #2, and #3 were 20.7 ± 21.5%, 18.7 ± 9.7%, 20.5 ± 10.1%. Performance was better in robotic mode: for track #1, #2, and #3, time percentage outside the track was 4.0 ± 7.3% (*p* = 0.010), 2.7 ± 3.6% (*p* < 0.001), and 3.3 ± 5.9% (*p* = 0.002). Similarly, robotic mode led to a significant decrease in the number of missed segments while traversing the track as compared to manual mode. For tracks #1, #2 and #3, it significantly reduced from 6.3 ± 4.7, 4.7 ± 2.9, and 3.8 ± 2.6 to 1.0 ± 1.61 (*p* = 0.007), 1.1 ± 1.57 (*p* = 0. 011), and 0.3 ± 0.4 (*p* = 0.002).

**Table 1:** Comparison of manual versus robotic modes

Scenarios	Parameters recorded during user study	Manual mode	Robotic mode	*p* value
Scenario-A	Duration to traverse the track			
	Track 1	84.2 ± 26.9 s	79.5 ± 29.4 s	*p* = 0.743
	Track 2	93.1 ± 38.3 s	60.3 ± 11.8 s	*p* = 0.026
	Track 3	80.7 ± 31.9 s	65.1 ± 22.1 s	*p* = 0.104
	Percentage of time spent outside the track			
	Track 1	20.7 ± 21.5%	4.0 ± 7.3%	*p* = 0.010
	Track 2	18.7 ± 9.7%	2.7 ± 3.6%	*p* < 0.001
	Track 3	20.5 ± 10.1%	3.3 ± 5.9%	*p* = 0.002
	Number of missed segments while traversing track			
	Track 1	6.3 ± 4.7	1.0 ± 1.61	*p* = 0.007
	Track 2	4.7 ± 2.9	1.1 ± 1.57	*p* = 0.011
	Track 3	3.8 ± 2.6	0.3 ± 0.4	*p* = 0.002
Scenario-B	Duration to traverse the track	232.2 ± 83.37 s	161.9 ± 42.9 s	*p* < 0.001
	Percentage of time spent outside the track	36.1 ± 19.1%	16.8 ± 11.1%	*p* < 0.001
	Number of missed segments while traversing track	8.1 ± 5.7	3.5 ± 2.8	*p* < 0.001

In Scenario-B, the robotic mode performed significantly better compared to the manual mode across the three recorded parameters (Fig. 5b and Table 1). The time to navigate the track while targeting the ulcer markers placed in the stomach lining under manual mode took significantly longer (232.27 ± 83.37 s) than the robotic mode (161.9 ± 42.9 s, *p* < 0.001). Similarly, the percentage of time for which the scope’s focus shifted outside the track was lower for manual mode (36.1 ± 19.1%) as compared to robotic mode (16.8 ± 11.1%, *p* < 0.001). Also, the number of missed segments for manual mode (8.1 ± 5.7) were lower compared to robotic mode (3.5 ± 2.8, *p* < 0.001).

The NASA-TLX scale reflected that the workload for robotic mode was lower as compared to manual mode (Fig. 5c). A lower score corresponds to a more favorable assessment for mental demand, physical demand, temporal demand, effort, and frustration level. In the case of performance, a lower score indicates a high level of proficiency. The NASA-TLX scores under manual mode were 6.3 ± 1.3 for mental demand, 6.9 ± 1.5 for physical demand, 4.9 ± 2.7 for temporal demand, 6.2 ± 2.1 for effort, 6.1 ± 2.5 for performance, and 6.0 ± 2.2 for frustration. Robotic mode exhibited significantly lower scores as compared to manual mode, except for temporal demand. The NASA-TLX scores under robotic mode were 4.8 ± 1.7 for mental demand (*p* = 0.034), 3.2 ± 1.6 for physical demand (*p* < 0.001), 4.4 ± 1.5 for temporal demand (*p* = 0.529), 4.4 ± 1.2 for effort (*p* = 0.027), 3.7 ± 1.6 for performance (*p* = 0.046), and 3.7 ± 2.0 for frustration (*p* = 0.047).

In addition to the user-study, the time required for installation and disassembly was also measured. The process of attaching the circular gears to the endoscope’s knob, positioning the endoscope onto the support plate, securing the hinges, and inserting it into the scope adapter took approximately one minute. Similarly, the disassembly process also required the same amount of time.

## Discussion

In the robotic mode under Scenario-A, the subjects preferred providing unidirectional step inputs (for example ‘left’ and ‘up’) to traverse diagonally in track #1 or along the curvature in track #3. This resulted in similar timing to that of manual mode. However, the percentage of time for which the scope’s focus shifts outside the track and the number of missed segments were significantly lower for robotic mode for all the three tracks. This validated the use of robotic mode to precisely navigate through tracks of 2 mm in width.

In the manual mode under Scenario-B, the percentage of time for which the scope’s focus shifts outside the track and the number of missed segments were higher due to (a) misalignment of the scope focus caused by abrupt manual insertion/retraction motion, (b) difficulty to hold the deflection knob stable at the intended position, (c) disorientation of the endoscope distal tip inside the stomach cavity. These factors collectively led to a prolonged effort to reorient the scope’s focus back onto the track after deflection, consequently resulting in an increase in the task duration. These challenges were addressed by the robotic mode. In this mode: (a) The operator was relieved from the burden of supporting the endoscope’s weight, (b) the adaptable speed control facilitated quicker movements on straight sections of the track while offering better control and slower motion on curved sections, and (c) the scope adapter maintained endoscope stability, preventing sudden unintended movements and focus misalignments. The NASA-TLX analysis also indicated that the subjects favored the robotic mode as compared to the manual mode providing a user-friendly control mechanism. Significant differences were observed for mental demand, physical demand, effort, performance, and frustration. For temporal demand, the subjects did not feel the time pressure for completing the tasks under two modes, and this resulted in similar scores.

**Fig. 6:**
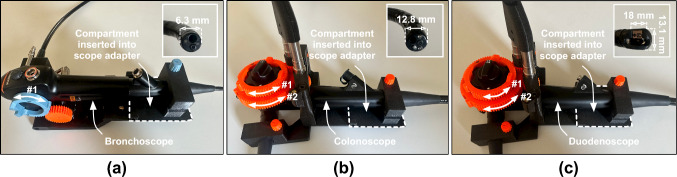
Support plates hosting **a** bronchoscope, **b** colonoscope, and **c** duodenoscope. The former support plate host mechanism to actuate up–down movement of the bronchoscope’s distal end. The latter support plate hosts a mechanism to enable both up–down and left–right movements of the distal ends of the bronchoscope and duodenoscope. Zoomed panels display the distal ends of each endoscope. The compartment inserted into the scope adapter (marked by a rectangular dotted line) is the same for all the support plates

This work demonstrates the advantage of extending the usage of scope adapter (originally built for a rigid zero, angulated [9, 11], and articulated scopes [10, 14]) for flexible endoscopes. The generic design enables utilizing the proposed system across different endoscopic procedures (such as bronchoscopy, colonoscopy, or duodenoscopy). For example, a new support plate could be designed to host a flexible bronchoscope (Fig. 6a). Though a new design of support plate (to actuate deflection knob) is required, the same design of the scope adapter (Fig. 2c) can be used to achieve insertion/retraction and the rotation of the bronchoscope along its shaft. In several endoscopes, such as colonoscope (Fig. 6b) and duodenoscope (Fig. 6c), the support plate designed for gastroscope (Fig. 2b) can be reutilized. This further underscores the value of using a modular system where the components can be configured to facilitate multiple types of usage. The proposed support plate and scope adapter design do not impede access to the buttons on the endoscope’s control section (Fig. 2d). The overall system can be connected to conventionally used air/CO_2_ insufflators, such as UCR (Olympus Medical Systems, Tokyo, Japan) or CO2MPACT Endoscopic Insufflator System (Steris, Mentor, U.S.A), and irrigation pumps. Alternatively, smart pressure control surgical insufflators such as EVA-15 (Palliare, Galway, Ireland) [15] and UHI-3 (Olympus Medical Systems, Tokyo, Japan) [16] can be integrated for a seamless clinical experience.

Several enhancements can be made to the current design of the proposed system to improve its applicability. First, the current version uses a rail to prevent buckling and guide the flexible endoscope during insertion/retraction. This increases the workspace of the setup in the operating room. To reduce the footprint of the proposed system, a highly compressible origami-based anti-buckling support sheath can be deployed [17]. Second, a mechanism comprising of push button switches could be used to enable pressing of air/water irrigation and suction buttons. Third, while a game controller was used as an input device, other human computer interfaces [18, 19], such as stylus/handle [6, 20], interfaces based on eye-gaze and/or head motion [7], can be integrated with the proposed system to provide actuation commands. While game controller and stylus/handle engages operator’s hands, head/eye tracking devices offer a hands-free solution for endoscope movements [11]. In addition, instead of displaying the view acquired from the endoscope on a 2D display screen, head mounted display devices can be used to render the view in virtual reality or mixed reality environment [21]. These devices also have inbuilt sensors to detect head pose and can provide actuation commands for the endoscope movement. Lastly, visual cues can also be rendered to compensate for the loss of tactile feedback. These visual cues could assist in depicting the endoscope shape (e.g. inserted length and deflection angles) based on the motor state, and the forces exerted on the lumen using fiber Bragg grating sensors [22].

The proposed system was trialed in an environment using basic endoscope manipulation techniques. To further assess the system, we plan to evaluate it in high-fidelity phantoms, such as a colon phantom during cecal intubation, where advanced techniques (e.g., hooking, left turn shortening, and right turn shortening [12]) can be employed to navigate in the presence of luminal tissue deformation. The user study was primarily conducted to demonstrate the ease of using the actuated system for maneuvering. Experienced endoscopists were not included as subjects, and they may perform as well as the proposed system [23]. However, robotic actuation offers several advantages over manual manipulation, including (a) superior endoscope stability, (b) effective alleviation of hand tremors and wrist discomfort associated with prolonged endoscope manipulation, and (c) reduced operator fatigue by enabling procedures to be performed comfortably while seated. These ergonomic benefits may prevent musculoskeletal pain issues reported by expert endoscopists [24]. Evaluating the system in clinical use case scenarios will be necessary to confirm this. Additionally, advanced guidance solutions that combine sensing, intelligent algorithms, and motor control for actuation can be used to automate the navigation.

In conclusion, the proposed system enhances the maneuverability of flexible endoscopes. Moreover, the simplified design eases the installation and disassembly of the system for usage. The user study validated the effectiveness of the proposed scope adapter and laid a strong foundation for future development of modular and generic scope assistant systems.

## References

[1] Pullens HJ, van der Stap N, Rozeboom ED, Schwartz MP, van der Heijden F, van Oijen MG, Siersema PD, Broeders IA (2016). Colonoscopy with robotic steering and automated lumen centralization: a feasibility study in a colon model. Endoscopy.

[2] Rozeboom E, Ruiter J, Franken M, Broeders I (2014). Intuitive user interfaces increase efficiency in endoscope tip control. Surg Endosc.

[3] Reilink R, de Bruin G, Franken M, Mariani MA, Misra S, Stramigioli S (2010) Endoscopic camera control by head movements for thoracic surgery. In: 2010 3rd IEEE RAS & EMBS international conference on biomedical robotics and biomechatronics, pp 510–515

[4] Ruiter J, Rozeboom E, van de Voort M, Bonnema M, Broeders I (2012) Design and evaluation of robotic steering of a flexible endoscope. In: 2012 4th IEEE RAS & EMBS international conference on biomedical robotics and biomechatronics (BioRob), pp 761–767

[5] Ruiter J, Bonnema GM, van der Voort MC, Broeders IAMJ (2013). Robotic control of a traditional flexible endoscope for therapy. J Robot Surg.

[6] Iwasa T, Nakadate R, Onogi S, Okamoto Y, Arata J, Oguri S, Ogino H, Ihara E, Ohuchida K, Akahoshi T (2018). A new robotic-assisted flexible endoscope with single-hand control: endoscopic submucosal dissection in the ex vivo porcine stomach. Surg Endosc.

[7] Sivananthan A, Kogkas A, Glover B, Darzi A, Mylonas G, Patel N (2021). A novel gaze-controlled flexible robotized endoscope; preliminary trial and report. Surg Endosc.

[8] Kume K, Sakai N, Goto T (2015). Development of a novel endoscopic manipulation system: the endoscopic operation robot ver. 3. Endoscopy.

[9] Khorasani M, Abdurahiman N, Padhan J, Zhao H, Al-Ansari A, Becker AT, Navkar N (2023). Preliminary design and evaluation of a generic surgical scope adapter. Int J Med Robot Comput Assist Surg.

[10] Abdurahiman N, Khorasani M, Padhan J, Baez VM, Al-Ansari A, Tsiamyrtzis P, Becker AT, Navkar NV (2023). Scope actuation system for articulated laparoscopes. Surg Endosc.

[11] Abdurahiman N, Padhan J, Zhao H, Balakrishnan S, Al-Ansari A, Abinahed J, Velasquez CA, Becker AT, Navkar NV (2022) Human-computer interfacing for control of angulated scopes in robotic scope assistant systems. In: 2022 international symposium on medical robotics (ISMR), IEEE, pp 1–7

[12] Lee SH, Park YK, Lee DJ, Kim KM (2014). Colonoscopy procedural skills and training for new beginners. World J Gastroenterol.

[13] Suresh KP (2011). An overview of randomization techniques: an unbiased assessment of outcome in clinical research. J Hum Reprod Sci.

[14] Abdurahiman N, Padhan J, Khorasani M, Zhao H, Baez VM, Al-Ansari A, Becker AT, Navkar NV (2023) Interfacing mechanism for actuated maneuvering of articulated laparoscopes using head motion. In: 2023 IEEE international conference on mechatronics and automation (ICMA), pp 2289–2296

[15] McInerney N, Khan MF, O’Malley K, McCormack O, Walsh T, Conneely J, Cahill R (2022). User evaluation of a novel smart insufflator for laparoscopic surgery-the EVA-15. Surg Technol Int.

[16] Nakajima K, Nishida T, Milsom JW, Takahashi T, Souma Y, Miyazaki Y, Iijima H, Mori M, Doki Y (2010). Current limitations in endoscopic CO2 insufflation for NOTES: flow and pressure study. Gastrointest Endosc.

[17] Sargent B, Butler J, Seymour K, Bailey D, Jensen B, Magleby S, Howell L (2020). An origami-based medical support system to mitigate flexible shaft buckling. J Mechan Robot.

[18] Hamza H, Baez VM, Al-Ansari A, Becker AT, Navkar NV (2023). User interfaces for actuated scope maneuvering in surgical systems: a scoping review. Surg Endosc.

[19] Shabir D, Anbatawi M, Padhan J, Balakrishnan S, Al-Ansari A, Abinahed J, Tsiamyrtzis P, Yaacoub E, Mohammed A, Deng Z, Navkar NV (2022). Evaluation of user-interfaces for controlling movements of virtual minimally invasive surgical instruments. Int J Med Robot.

[20] Shabir D, Abdurahiman N, Padhan J, Anbatawi M, Trinh M, Balakrishnan S, Al-Ansari A, Yaacoub E, Deng Z, Erbad A, Mohammed A, Navkar NV (2022). Preliminary design and evaluation of a remote tele-mentoring system for minimally invasive surgery. Surg Endosc.

[21] Mak YX, Zegel M, Abayazid M, Mariani MA, Stramigioli S (2022) Experimental evaluation using head motion and augmented reality to intuitively control a flexible endoscope. In: 9th IEEE RAS/EMBS international conference for biomedical robotics and biomechatronics (BioRob), pp 1–7

[22] Lu Y, Lu B, Li B, Guo H, Liu Yh (2021). Robust three-dimensional shape sensing for flexible endoscopic surgery using multi-core FBG sensors. IEEE Robot Autom Lett.

[23] Lu Y, Wei R, Li B, Chen W, Zhou J, Dou Q, Sun D, Liu YH (2023) Autonomous intelligent navigation for flexible endoscopy using monocular depth guidance and 3-D shape planning. In: 2023 IEEE international conference on robotics and automation (ICRA), pp 1–7

[24] Harvin G (2014). Review of musculoskeletal injuries and prevention in the endoscopy practitioner. J Clin Gastroenterol.

